# A glimpse into the pipeline of anti-obesity medication development: combining multiple receptor pathways

**DOI:** 10.3389/fendo.2025.1630199

**Published:** 2025-09-11

**Authors:** Christine Park, Yushin Kim, Sawye Raygani, Eduardo Grunvald

**Affiliations:** ^1^ Department of Medicine, University of California San Diego, La Jolla, CA, United States; ^2^ Saint Louis University School of Medicine, Saint Louis, MO, United States; ^3^ University of California San Diego School of Medicine, La Jolla, CA, United States

**Keywords:** obesity, anti-obesity pharmacotherapy, emerging therapies, obesity drug development, GLP-1 receptor agonists, combination therapy

## Abstract

Obesity has been historically a stubborn chronic metabolic disease, resistant to multiple therapeutic modalities. Although effective in the short-term for some people, lifestyle interventions have proven difficult to maintain in the long-term. Metabolic bariatric surgery is the most effective treatment for durable weight loss and improvement of obesity-related conditions but is invasive and vastly underutilized. For decades, patients and clinicians confronted a wide gap between lifestyle modification and bariatric procedures. Anti-obesity pharmacotherapy was plagued by either safety concerns or very modest effectiveness. Recently, the availability of highly effective medications has given patients living with obesity hope for better health. These advances represent a culmination of many years of scientific progress regarding our understanding of human weight regulation and the beginning of a new era in treating metabolic diseases. In fact, many molecules are under investigation for obesity therapy, some with novel mechanisms. Since data on these putative agents are appearing at accelerated speed, the aim of this review is to provide an updated synopsis of emerging agents, highlighting the correlation between efficacy and combination strategies.

## 1 Introduction

Obesity is linked to a multitude of chronic conditions, including type 2 diabetes (T2D), cardiovascular disease, cancer, and hypertension, among many others. From a global epidemiologic perspective, it is associated with increased morbidity and mortality, healthcare costs, absenteeism, and presenteeism (1). Of major concern, despite a long history of public health efforts, rates of global obesity, childhood obesity, and severe obesity have risen steadily (2). In the United States (US), it is estimated that 50% of the adult population will have obesity by 2030 (3). Economic analyses suggest that direct medical costs attributed to obesity among adults in the US are over $260 billion (4).

Evidence-based guidelines provide recommendations to healthcare professionals (HCP) for intense lifestyle interventions (ILI) with the aim of weight reduction and mitigation of cardiovascular risk (5). Unfortunately, multicomponent programs are often unreimbursed and poorly accessible, especially for individuals of lower socioeconomic status, who have disproportionate rates of obesity (3). Moreover, even the most intense, high-quality strategies do not result in long-term weight loss (6). Participants often find it difficult to continue with the healthy habits practiced during the intervention, especially without continued support for maintenance. Although such programs may help people achieve clinically meaningful weight reduction in the short-term, the modest effects have not translated into significant cardiovascular benefit (7).

Metabolic bariatric surgery (MBS) stands out as the most effective treatment for severe obesity, demonstrating significant and sustained weight loss while also improving various obesity-related health complications (8). Until recently, it was the only weight loss intervention to show reductions in mortality (9, 10). Despite its efficacy, the number of individuals opting for bariatric surgery remains low, with less than 1% of the eligible population receiving these procedures in the US (11). For decades, there has been a substantial therapeutic gap between lifestyle interventions and MBS.

The development of safe and effective pharmacological agents for obesity has encountered significant challenges, marked by the emergence and subsequent decline of many medications due to adverse side effects, such as cardiovascular risk (12, 13). The past decade, however, has witnessed a resurgence in the development and approval of anti-obesity medications (AOM). In the US, aside from phentermine and orlistat, approved by the Food and Drug Administration (FDA) in 1959 and 1999, respectively, phentermine/topiramate extended release (Qsymia^®^) was approved in 2012. In 2014, naltrexone/bupropion sustained release (Contrave^®^) and liraglutide 3 mg (Saxenda^®^) were approved for chronic weight management. Adoption of these agents was underwhelming, however (14). These drugs, spanning diverse mechanistic pathways, have failed to achieve a reduction in mean body weight exceeding 10% (13, 15). Semaglutide 2.4 mg (Wegovy^®^) and tirzepatide (Zepbound^®^), approved for the treatment of obesity in 2021 and 2024, respectively, produced corresponding mean weight loss of 17% and 23% in clinical trials of subjects with obesity, but without T2D (16, 17). Given the efficacy and safety of these novel agents, utilization soared (18). Until recently, demand far outweighed supply in several markets, contributing to access challenges and fragmented treatment (19). However, the cost of these medications and coverage exclusion by many payors in the US results in access challenges for many patients that could benefit. Additionally, many public healthcare insurance programs do not reimburse for AOMs, further exacerbating access inequalities. Despite this, it is thought that this new generation of therapeutics represents a historical inflection point in the advancement for improving obesity and related chronic metabolic diseases. Moreover, compared to older medications, these agents show more weight loss in individuals with T2D, a population which is associated with inferior outcomes across many treatment modalities (20).

It is not a coincidence that AOMs are demonstrating progressively superior effectiveness. The adipocyte hormone leptin was discovered in 1994 (21). Since then, our understanding of the metabolic, cellular, and molecular physiology of human weight regulation has expanded greatly. These discoveries have further established obesity as a chronic, progressive biological disease requiring physiologic treatments beyond willful energy restriction and expenditure alone. Additionally, genetic predisposition plays a role, influencing metabolic processes and fat storage propensity (22). A negative energy balance lowers metabolic rate and intensifies hunger and cravings (23, 24). The central nervous system (CNS) is at the center of integration and regulation of long-term energy stores (adipose mass). Multiple brain areas, with genetic determinants, are involved in the control of ingestive behaviors, including centers that not only modulate satiety, but also those responsible for sensory inputs, learning, memory, emotions, cognitive control, and hedonic behaviors (25). The CNS mediates metabolic processes that regulate weight in response to peripheral signals derived from nutrients, hormones, and neurological inputs. Given that the gastrointestinal (GI) tract contains the sentinel tissues to first encounter ingested nutrients, many of these signals are gut peptides that inform the brain, either through stimulated hormones or through the vagus nerve, a connection known as the gut-brain axis. These hormones include glucagon-like peptide 1 (GLP-1), glucose dependent insulinotropic polypeptide (GIP), oxyntomodulin, and peptide tyrosine-tyrosine (PYY) secreted from intestinal endocrine cells, and the pancreatic hormones glucagon and amylin (26). They peak postprandially and are considered “metabolic switches”, involved in shifting metabolic processes from the fasting to the fed state (27). Detailed reports on cellular and molecular physiologic mechanisms for specific agents have been published (28).

Endogenous gut hormones however, are enzymatically metabolized within a few minutes (29). These peptides have been modified with fatty acid moieties to promote albumin binding, thereby extending their half-lives significantly for pharmacotherapeutic effectiveness (30). Not surprisingly, these advances have resulted in more targeted pharmacological strategies. Despite the array of available medications, there remains an urgent and ongoing effort to formulate novel drugs that comprehensively address the unmet needs related to heterogeneity of obesity and response to a given therapy. Since neuro-enteroendocrine pathways are redundant and coordinated, targeting more than one of these signals results in more potent and synergistic effects on weight loss outcomes. The clinical rationale for this strategy, utilized in other chronic metabolic diseases, is manifested in established therapies like combining phentermine and topiramate, or bupropion and naltrexone. Using a more refined tactic, unimolecular agents can stimulate more than one receptor, augmenting desired therapeutic outcomes, tirzepatide being a prime example. In more refractory cases, drugs with varied mechanisms of action (MOA) in combination are used in clinical practice (31). Notably, all biologic therapies for obesity should be used as adjuncts to optimal lifestyle modification. Obesity is a result of an obesogenic environment exerting its effects in genetically predisposed individuals, manifested as poor nutrition, reduced physical activity, and maladaptive behaviors. AOMs can help patients overcome biological forces that promote unhealthy dietary habits. Pharmacotherapy can produce even better results when used together with more intense lifestyle interventions, especially with older generation medications (32–34). Moreover, even with the use of these therapies, patients need to be appropriately supported so that nutrition is optimized, muscle mass is maintained, and nutritional deficiencies are prevented. The ultimate combination treatment is applying AOMs before and/or after MBS (35–37).

**Figure 1:**
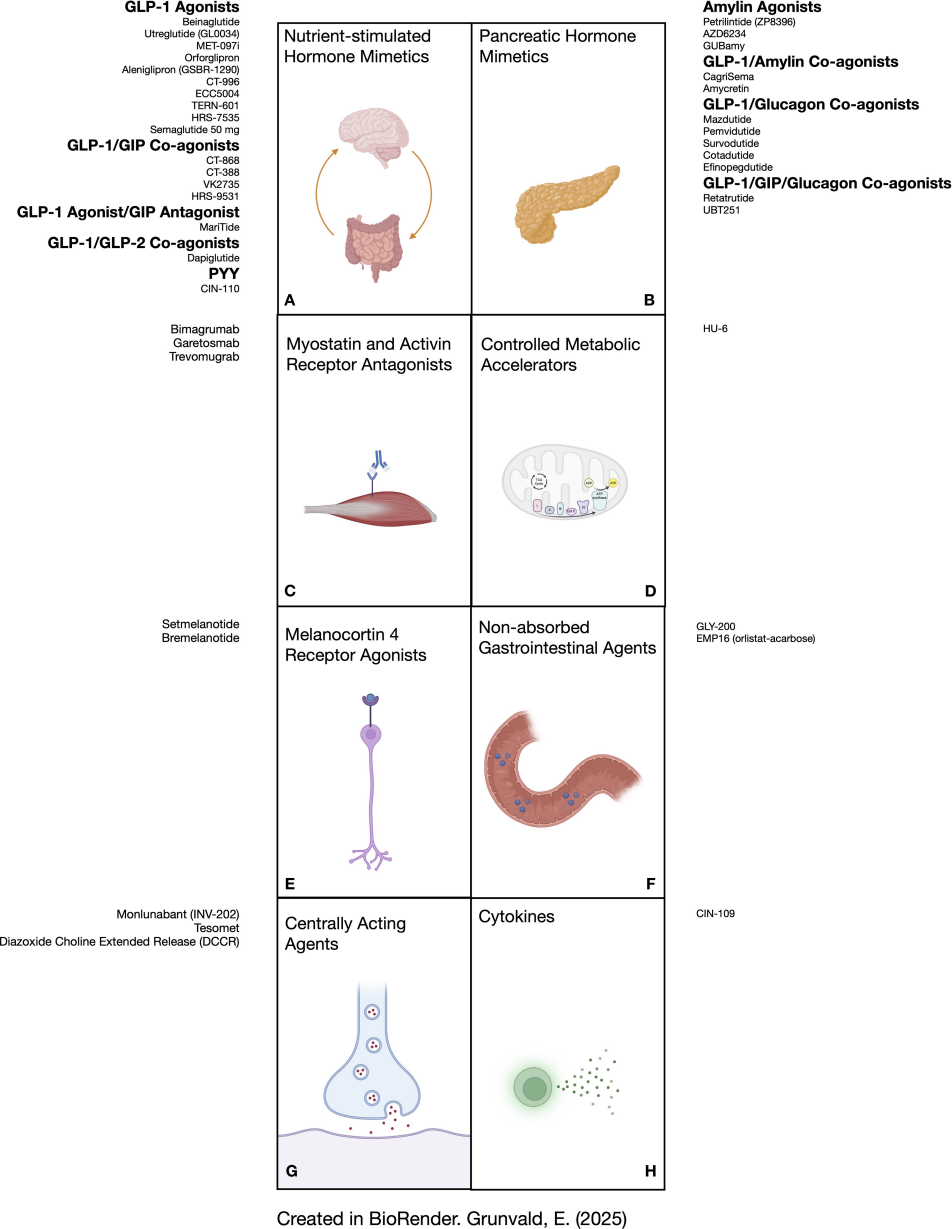
Depiction of anti-obesity medications in development according to their physiologic mechanism of action. **(A)** shows molecules that stimulate receptors for GLP-1 as monotherapy and in combination with other gut hormones (GIP, GLP-2, and PYY). **(B)** lists molecules that activate receptors for pancreatic hormones (amylin, glucagon), some in combination with incretin hormones. **(C)** shows antibodies that bind to activin receptors (bimagrumab and garetosmab) or myostatin receptors (trevomugrab) to preserve skeletal muscle mass. **(D)** illustrates a controlled metabolic accelerator. **(E)** represents melanocortin-4 receptor agonists. **(F)** shows agents that function through mechanisms in the gut lumen without significant absorption. **(G)** lists molecules that exert their effects primarily in the central nervous system. **(H)** shows an example of a cytokine analogue that can promote satiety and lipolysis. GIP, glucose dependent insulinotropic polypeptide; GLP-1, glucagon-like peptide 1; GLP-2, glucagon-like peptide 2; PYY, peptide tyrosine tyrosine.

The aim of this narrative review is to provide the most up to date presentation of molecules under development for the chronic treatment of obesity. Because the presentation of data related to emerging progress in this space changes literally from week to week, the purpose of this report is to provide the reader a view into the world of future therapies. As such, the present review includes all publicly available human data and is not restricted exclusively to peer-reviewed publications. We only include drugs in development that have reported effects on weight loss. **Figure 1** shows the various agents presented in this report and their corresponding mechanistic groups.

## 2 Methods

For this narrative review, we searched PubMed, clinicaltrials.gov, conference proceedings, pharmaceutical websites, and press releases from June 2008 through June 2025. The search terms used to identify emerging medications for obesity treatment included: ‘obesity,’ ‘pharmacotherapy,’ ‘anti-obesity medications’, and ‘clinical phases’. Pipeline drugs, defined as pharmaceuticals for chronic weight management in development that have not yet been approved for clinical use, were included in the narrative review if human trial data were publicly available with sufficient information on the drug’s time of development, clinical phase, MOA, dosing information, and reporting on weight loss outcomes. Preclinical data were excluded. We included only data for adult subjects unless studies pooled adult and pediatric participants for the therapy of rare genetic or syndromic obesity. Data were excluded if the development on a particular agent was terminated. Descriptions of adverse effects are beyond the scope of this report. **Tables 1** and **2** list the agents reviewed with their MOA, phase of development, trial data, and associated biopharmaceutical companies, for target populations with and without T2D, respectively.

**Table 1:** Agents reviewed with their MOA, phase of development, trial data, and associated biopharmaceutical companies, for target populations with type 2 diabetes.

Molecule	Mechanism of action	Highest phase of investigation	Duration of observation (wks)	Efficacy (mean total weight loss)	Route of administration	Biopharmaceutical companies	Reference
Aleniglopron	GLP-1 RA	2	12	3.2%	PO	Structure Therapeutics	(51)
Beinaglutide	GLP-1 RA	3	12	6.0%	SC	Shanghai Benemae	(40)
Bimagrumab	Activin Receptor Antagonist	2	48	6.5%	IV	Versanis Bio*, Eli Lilly	(135)
CagriSema	GLP-1/Amylin	3	68	13.7%	SC	Novo Nordisk	(77)
Cotadutide	GLP-1/Glucagon	2	48	3.3%	SC	AstraZeneca	(102)
CT-868	GLP-1/GIP	2	26	5.7%	SC	Carmot Therapeutics*, Roche	(60, 61)
Efinopegdutide	GLP-1/Glucagon	2	24	8.5%	SC	Merck	(104)
GLY-200	Mucin-complexing Polymer	NA	2	1.8%	PO	Glyscend Therapeutics	(150–152)
HRS-9531	GLP-1/GIP	2	20	7.1%	SC	Kailera	(67)
MariTide	GLP-1 RA/GIP antagonist	2	52	17%	SC	Amgen	(71)
Mazdutide	GLP-1/Glucagon	2	20	7.1%	SC	Innovent	(90)
Orforglipron	GLP-1 RA	3	40	7.9%	PO	Eli Lilly	(48)
Pemvidutide	GLP-1/Glucagon	1	12	7.7%	SC	Altimmune	(93)
Retatrutide	GLP-1/GIP/Glucagon	2	36	16.9%	SC	Eli Lilly	(106)
Semaglutide 50 mg	GLP-1 RA	3	52	8.0%	PO	Novo Nordisk	(57)
Survodutide	GLP-1/Glucagon	2	48	13%	SC	Zealand Pharma*, Boehringer Ingelheim	(98)
Tesomet	Monoamine Reuptake Inhibitor	NA	12	3%	PO	Saniona	(143)

*Primary company involved in research and development of the molecule.

GIP, glucose dependent insulinotropic polypeptide; GLP-1, glucagon-like peptide 1; IV, intravenous; NA, not applicable; PO, per os; RA, receptor agonist; SC, subcutaneous.

**Table 2:** Agents reviewed with their MOA, phase of development, trial data, and associated biopharmaceutical companies, for target populations without type 2 diabetes.

Molecule	Mechanism of action	Highest phase of investigation	Duration of observation (wks)	Efficacy (mean total weight loss)	Route of administration	Biopharmaceutical companies	Reference
Aleniglopron	GLP-1 RA	2	12	6.2%	PO	Structure Therapeutics	(51)
Amycretin	GLP-1/Amylin	1	16	13%	PO	Novo Nordisk	(82)
Amycretin	GLP-1/Amylin	2	36	24.3%	SC	Novo Nordisk	(83)
Bremelanotide^‡^	MC4R agonist	2	8	4.4%	SC	Palatin	(128)
CagriSema	GLP-1/Amylin	3	68	22.7%	SC	Novo Nordisk	(76)
CIN-109	GDF-15	1	6	3.7%	SC	CinFina Pharma	(117)
CIN-110	PYY	1	1	1.8%	SC	CinFina Pharma	(114)
CT-388	GLP-1/GIP	1	4	8.5%	SC	Carmot Therapeutics,* Roche	(60, 63)
CT-996	GLP-1 RA	1	4	7.3%	PO	Carmot Therapeutics,* Roche	(52)
Dapiglutide	GLP-1/GLP-2	2	12	4.3%	SC	Zealand Pharma	(113)
DCCR	Central Anorectic	3	52	-3.6%^§^	PO	Soleno Therapeutics	(147)
ECC5004	GLP-1 RA	1	4	5.8%	PO	Eccogene, AztraZeneca	(53)
EMP16	Glucosidase and Lipase Inhibitor	2	26	6.3%	PO	Empros Pharma	(154)
GUBamy	Amylin	1	6	3.3%	SC	Gubra	(85)
HRS-7535	GLP-1 RA	1	4	6.5%	PO	Shandong Suncadia Medicine Co.	(55)
HRS-9531	GLP-1/GIP	2	24	16.8%	SC	Kailera	(68)
HU-6	CMA	2	8	2.5%	PO	Rivus Pharmaceuticals	(121)
MariTide	GLP-1 RA/GIP antagonist	2	52	20%	SC	Amgen	(71)
Mazdutide	GLP-1/Glucagon	3	48	12.6%	SC	Innovent	(91)
MET-097i	GLP-1 RA	2	12	11.3%	SC	Metsera	(44)
Monlunabant	CB-1 receptor inverse agonist	2	16	6.4%	PO	Inversago Pharma*, Novo Nordisk	(119)
Orforglipron	GLP-1 RA	2	36	14.7%	PO	Eli Lilly	(45)
Pemvidutide	GLP-1/Glucagon	2	48	15.6%	SC	Altimmune	(93)
Petrelintide	Amylin	1	16	8.3%	SC	Zealand Pharma*, Roche	(81)
Retatrutide	GLP-1/GIP/Glucagon	2	48	24.2%	SC	Eli Lilly	(105)
Semaglutide 25 mg	GLP-1 RA	3	64	13.6%	PO	Novo Nordisk	(59)
Semaglutide 50 mg	GLP-1 RA	3	68	15.1%	PO	Novo Nordisk	(58)
Setmelanotide	MC4R agonist	2	40	12.4%	SC	Rhythm Pharmaceuticals	(126)
Survodutide	GLP-1/Glucagon	2	46	18.7%	SC	Zealand Pharma*, Boehringer Ingelheim	(96)
TERN-601	GLP-1 RA	1	4	5.5%	PO	Terns Pharmaceuticals	(54)
Tesomet^Δ^	Monoamine Reuptake Inhibitor	2	13	6.8%	PO	Saniona	(144)
Tesomet^†^	Monoamine Reuptake Inhibitor	2	24	6.6%	PO	Saniona	(145)
UBT251	GLP-1/GIP/Glucagon	1	12	15.1%	SC	United Biotechnology*, Novo Nordisk	(110)
Utreglutide	GLP-1 RA	1	8	10.7%	SC	Sun Pharma	(41)
VK2735	GLP-1/GIP	2	13	14.7%	SC	Viking Therapeutics	(65)
VK2735	GLP-1/GIP	1	4	5.3%	PO	Viking Therapeutics	(65)

^‡^In combination with tirzepatide.

*Primary company involved in research and development of the molecule.

^§^Weight gain attributed to increase in lean mass.

^Δ^Data reported for participants with Prader-Willi Syndrome.

^†^Data reported for participants with hypothalamic obesity.

CB-1, cannabinoid 1; CMA, controlled metabolic accelerator; GDF-15, growth differentiation factor 15; GIP, glucose dependent insulinotropic polypeptide; GLP-1, glucagon-like peptide 1; GLP-2, glucagon-like peptide 2; IV, intravenous; MC4R, melanocortin 4 receptor; NA, not applicable; PO, per os; PYY, peptide tyrosine tyrosine; RA, receptor agonist; SC, subcutaneous.

## 3 Nutrient-stimulated hormone (NuSH) therapies

**Figure 2:**
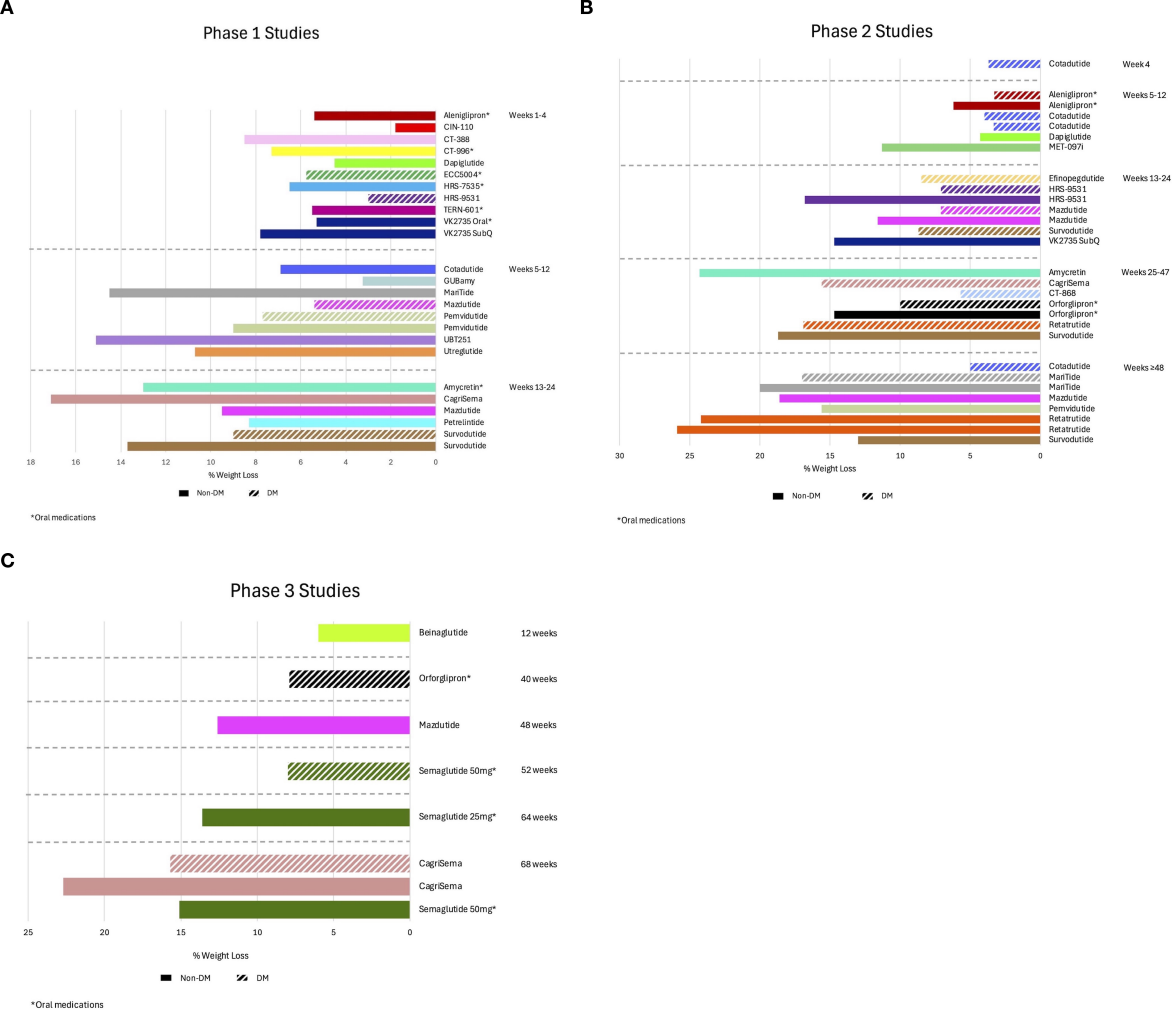
Observed weight loss of nutrient-stimulated hormone (NuSH) mimetics in development organized by trial phase and duration of studies. The graphs show mean weight loss outcomes separated by categorical duration of studies on the y-axis for phase 1 **(A)**, phase 2 **(B)**, and phase 3 **(C)** studies. Solid bars reflect studies for subjects without type 2 diabetes and bars with slashes for subjects with type 2 diabetes. DM, diabetes mellitus.

**Figures 2A-C** show the mean weight loss outcomes segmented in their investigation phases and periods of observation.

### 3.1 GLP-1 receptor agonist monotherapy

#### 3.1.1 Subcutaneous

##### 3.1.1.1 Beinaglutide

Beinaglutide is a GLP-1 receptor agonist (RA) that is administered subcutaneously three times a day with meals. A retrospective real-world study with an observation period of 3 months in a Chinese population with T2D shows a mean weight loss of 12.9% in patients treated with both insulin and beinaglutide without a control comparator group (38). A 12-week prospective randomized trial comparing beinaglutide with metformin in subjects with overweight or obesity, but without T2D, shows a mean weight loss of 9.5% and 5.1%, respectively (39). A phase 3 trial over 12 weeks, also in Chinese individuals without T2D, investigating beinaglutide 0.2 mg reveals a mean weight loss of 6% compared to 2.4% in the placebo group (40).

##### 3.1.1.2 Utreglutide (GL0034)

Utreglutide is a once-weekly GLP-1 RA administered subcutaneously (41). An 8-week phase 1 multiple ascending dose (MAD) study was conducted on healthy individuals with body mass index (BMI) 18–28 kg/m^2^ at doses of 450 mcg for 4 weeks (cohort 1), 900 mcg for 4 weeks (cohort 2), and an 8-week dose escalation up to 1,520 mcg (cohort 3) (41). Cohorts 1, 2, and 3 all show a significant reduction in body weight compared to baseline levels, with a mean reduction of 4.5%, 5.8%, and 10.7%, respectively, compared to 2.2% weight gain for the placebo group. In subjects with BMI ≥ 30 kg/m^2^, but without T2D, a phase 1 single ascending dose (SAD) trial shows that GL0034 at doses of 1,520 mcg, 2,000 mcg, and 2,520 mcg results in mean weight loss of 2.1%, 1.9%, and 2.5%, respectively, versus 0.3% weight gain for the placebo group one week after administration (42).

##### 3.1.1.3 MET-097i

MET-097i is an “ultra-long” acting GLP-1 RA with an elimination half-life of 380 hours (43). In a phase 2a study investigating weekly dosing in subjects without T2D, placebo-adjusted weight loss up to 11.3% is reported at 12 weeks (44). More details are not yet available. Due to its long half-life, it is also being considered for monthly dosing.

#### 3.1.2 Oral

##### 3.1.2.1 Orforglipron

Orforglipron is a small molecule non-peptide oral GLP-1 RA administered once daily. A phase 2 study in subjects having BMI ≥27 kg/m^2^ without T2D finds a mean body weight loss ranging from 9.4% to 14.7% across four doses (12, 24, 36, or 45 mg), versus 2.3% with placebo at 36 weeks (45). In another 26-week phase 2 study in participants with T2D and BMI ≥23 kg/m^2^, orforglipron at the maximum dose of 45 mg produces a statistically significant glycated hemoglobin (HbA1c) reduction of 2.1%, versus 0.4% with placebo and 1.1% with dulaglutide 1.5 mg (46). Mean body weight loss ranges from 9.6-10.0% at the three highest doses versus 2.2% and 4.0% for placebo and dulaglutide, respectively. Of note, pharmacokinetics of orforglipron is not significantly affected by food consumption, suggesting prandial restrictions may not be necessary for oral administration (47).

Results from the 40-week phase 3 ACHIEVE-1 trial in participants with obesity and poorly controlled T2D shows mean weight loss of 4.7%, 6.1%, 7.9% and 1.6% for groups receiving 3 mg, 12 mg, 36 mg, and placebo, respectively (48). The corresponding mean reductions in HbA1c percentage points are 1.3, 1.6, 1.5, and 0.1.

##### 3.1.2.2 Aleniglipron (GSBR-1290)

Aleniglipron is an oral small molecule GLP-1 RA. A 28-day phase 1b MAD study in participants with overweight or obesity demonstrates safety and tolerability at daily doses of 30 mg, 60 mg, and 90 mg with mean weight loss of 1.6%, 5.2%, and 5.4%, respectively, compared to 0.5% for placebo (49). Data from 12-week phase 2a studies are reported in subjects with BMI 27–40 kg/m^2^. The first study in participants with T2D shows a mean HbA1c reduction of 0.8% for both the 45 and 90 mg doses, compared to an increase of 0.2% in the placebo group (50). Corresponding mean weight loss is 3.3% and 3.2%, and 0.04% weight gain for the placebo group. The trial in individuals without T2D reveals a mean weight loss of 6.2% with 120 mg versus no change in the placebo group. A 36-week phase 2b study investigating approximately 300 subjects with overweight or obesity is planned (51).

##### 3.1.2.3 CT-996

CT-996 is a small molecule signal-biased GLP-1 RA administered daily by oral route. A 4-week phase 1 study investigated CT-996 in participants with BMI ≥30 kg/m^2^ in 3 different cohorts, with target doses of 90 mg and 120 mg (52). Cohorts 2 and 3 had the same target dose of 120 mg, but with lower intermediate titration doses for the latter group. Mean weight loss is 2.3%, 5.8%, and 7.3%, respectively, compared to 1.2% in the placebo arm.

##### 3.1.2.4 ECC5004

ECC5004 is a small molecule oral GLP-1 RA taken once daily. A 28-day phase 1 MAD study conducted in participants with T2D reveals mean weight loss of 3.3%, 3.6%, 4.9%, and 5.8%, corresponding to doses of 5 mg, 10 mg, 30 mg, and 50 mg (53).

##### 3.1.2.5 TERN-601

TERN-601 is another small molecule GLP-1 RA administered daily by oral route. A 28-day phase 1 MAD study was conducted in participants who were overweight or obese, revealing mean weight loss of 0.6%, 2.5%, 4.4%, and 5.5%, corresponding to groups administered placebo, 240 mg, 500 mg, and 740 mg (54).

##### 3.1.2.6 HRS-7535

HRS-7535 is a small molecule GLP-1 RA administered orally once a day. Over 29 days in a phase 1 trial with healthy volunteers, those in the treatment group demonstrate a mean weight loss of 6.5%, which is statistically superior to placebo (not reported) (55).

##### 3.1.2.7 Semaglutide 50 mg

Oral semaglutide is currently available up to a maximum dose of 14 mg for the treatment of T2D (56). High dose oral semaglutide for obesity has been investigated up to a maximum dose of 50 mg daily. In participants with poorly controlled T2D and BMI ≥25 kg/m^2^, a 52-week phase 3 randomized trial shows a mean weight loss of 4.4%, 6.7%, and 8.0% at doses of 14 mg, 25 mg, and 50 mg, respectively (57). Corresponding reductions in HbA1c are 1.5%, 1.8%, and 2.0%. The phase 3 OASIS 1 trial investigated high dose oral semaglutide in participants with overweight or obesity, but without T2D (58). At 68 weeks, mean weight loss is 15.1% at 50 mg compared to 2.4% for placebo. The 64-week OASIS 4 trial reports outcomes in a similar population using a maximum dose of 25 mg, which produces a mean weight loss of 13.6% compared to 2.2% for placebo (59).

### 3.2 GLP1/GIP receptor agonists

#### 3.2.1 Subcutaneous

##### 3.2.1.1 CT-868

CT-868 is a signaling biased dual GLP-1/GIP receptor co-agonist administered daily as a subcutaneous injection (60). A phase 2 trial in adults with T2D and BMI ≥27 kg/m^2^ demonstrates a mean HbA1c reduction of 2.0% at the 4.0 mg dose compared to an increase of 0.3% for placebo (61). Correspondingly, mean weight reduction is 5.7% versus 2.3% at 26 weeks. A 16-week phase 2 study investigating CT-868 in subjects with overweight or obesity and type 1 diabetes is in progress (62).

##### 3.2.1.2 CT-388

CT-388 is another signaling biased dual GLP-1/GIP receptor co-agonist administered weekly by subcutaneous route (61). A 4-week phase 1 MAD study in adults with overweight or obesity reveals a mean weight loss of 4.8% and 8.5% at maximum doses of 5 mg and 12 mg, respectively, compared to 0.8% in the placebo group (63).

##### 3.2.1.3 VK2735 subcutaneous

VK2735 is a dual GLP-1/GIP receptor co-agonist that is administered weekly by subcutaneous injection (64). A 28-day, phase 1 MAD trial results in dose-dependent mean body weight reductions of 2.5%, 3.7%, 4.1%, 7.4%, and 7.8% at target doses of 0.5 mg, 1.5 mg, 5.0 mg, 7.5 mg, and 10 mg, respectively, compared to 1.8% for the placebo group (65). The same study finds that 21 days following the last administration of VK2735, the differences in weight loss compared to placebo are maintained or improved. The VENTURE Study, a phase 2a parallel cohort trial, investigating doses of 2.5 mg, 5.0 mg, 10 mg and 15 mg over 13 weeks, shows mean weight loss of 9.1%, 10.9%, 12.9%, and 14.7%, respectively, compared to 1.7% for the placebo group (65).

##### 3.2.1.4 HRS-9531

HRS-9531 is a GLP-1 and GIP receptor co-agonist administered weekly by subcutaneous route. In a 4-week phase 1 study using a MAD design in participants with T2D, comparing treatment against dulaglutide and placebo, target doses of 0.3 mg, 1.5 mg, and 4.5 mg achieve mean weight loss of 0.3%, 2.6%, and 3.0%, respectively, with 2.2% mean weight loss for the dulaglutide group and 1.0% for placebo (66). A phase 2 study in participants with uncontrolled T2D over 20 weeks at target doses of 1.0 mg, 2.0 mg, 3.0 mg, and 4.5 mg reveals mean weight loss of 3.1%, 4.9%, 5.5%, and 7.1%, respectively, compared to 0.6% for the placebo group (67). Mean reductions in HbA1c percentage points are 2.1, 2.6, 2.7, 2.5, and 0.3, respectively. Similarly, another phase 2 trial in subjects with obesity but no T2D examined doses of 1.0 mg, 3.0 mg, 4.5 mg, and 6.0 mg over 24 weeks. Corresponding mean weight loss is 5.4%, 13.4%, 14.0%, and 16.8%, compared to 0.1% for placebo (68).

#### 3.2.2 Oral

##### 3.2.2.1 VK2735 Oral

VK2735 is also being developed as an oral agent administered daily. A 28-day, MAD phase 1 trial shows mean weight loss of 2.1%, 0.3%, 0.9%, 1.1%, 3.2% and 5.3% for placebo and target doses of 2.5 mg, 5 mg, 10 mg, 20 mg, and 40 mg, respectively (65).

### 3.3 GLP1 receptor agonist/GIP receptor antagonist

#### 3.3.1 MariTide

MariTide (maridebart cafraglutide, formerly AMG 133) is a GLP-1 receptor agonist conjugated to a human monoclonal antibody GIP receptor antagonist, administered subcutaneously once every four weeks (69). Although the mechanism is not completely understood, GIP receptor blockade has seemingly similar effects on reduction of food intake as agents with GIP receptor activation. A phase 1 trial enrolling participants with BMI 30–40 kg/m^2^ and without T2D shows that when given in multiple ascending doses, the mean body weight losses by day 85 range from 7.2% to 14.5% at the lowest (140 mg) and highest (420 mg) doses, respectively, compared to weight gain of 1.5% for the placebo group (70). Notably, weight loss is largely maintained for the two higher doses of 280 mg and 420 mg at 150 days after the last administration. A phase 2 52-week study reports a mean weight loss of 17% and 20% in participants with and without T2D, respectively (71). The weight loss trajectory is not plateaued at the 52-week time point. A second extension phase examining different maintenance doses and dosing frequencies is in progress.

### 3.4 Amylin receptor agonists

#### 3.4.1 Cagrilintide-semaglutide (CagriSema)

Cagrilintide is a long-acting amylin analogue administered subcutaneously once a week, with agonist properties for amylin and calcitonin receptors (72). When studied as monotherapy, cagrilintide produces a mean weight loss of 10.8% at the maximum dose of 4.5 mg over a period of 26 weeks (73). When combined with semaglutide 2.4 mg in a phase 1b clinical trial, a mean weight loss of 17.1% is observed at a dose of 2.4 mg over 20 weeks, compared to 9.8% with placebo and semaglutide (74). Glycemic parameters improved in all groups. In a 32-week phase 2 trial investigating the effect of CagriSema on HbA1c in subjects with T2D and BMI ≥ 27 kg/m^2^, investigational drug is superior to cagrlintide 2.4 mg alone (-2.2% v -0.9%), but not compared to semaglutide 2.4 mg alone (-1.8%) (75). In this study, CagriSema is superior to both cagrilintide and semaglutide for mean weight loss (15.6% v 8.1% v 5.1%, respectively).

Two 68-week phase 3 trials, REDEFINE-1, investigating CagriSema in subjects with obesity but not T2D, and REDEFINE-2, for individuals with obesity and T2D, have recently been completed. In REDEFINE-1, CagriSema (2.4 mg/2.4 mg) is compared to cagrilintide 2.4 mg, semaglutide 2.4 mg, and placebo, with corresponding mean weight loss of 22.7%, 11.8%, 16.1%, and 2.3% (76). For REDEFINE-2, CagriSema (2.4 mg/2.4 mg) is compared to placebo, with mean weight loss results of 13.7% and 3.4%, respectively (77).

#### 3.4.2 Petrelintide (ZP8396)

Petrelintide is a long-acting amylin analog that is administered subcutaneously once a week. A single dose phase 1 trial reveals mean weight loss of 2.6%, 3.6%, and 4.2% with doses of 0.7 mg, 1.7 mg, and 2.4 mg, respectively, one week after dose administration, compared to a gain of 0.6% for the placebo group (78). A 6-week phase 1b MAD trial finds that participants experience a 5.3% and 5.1% mean weight loss from baseline after being treated with 0.6 mg and 1.2 mg, respectively, *vs* 0.4% for those exposed to placebo (79, 80). Part 2 of this trial included extension over 16 weeks at doses of 2.4 mg, 4.8 mg, and 9.0 mg, resulting in mean weight loss of 4.8%, 8.6%, and 8.3%, respectively, compared to 1.7% for the placebo group (81). Phase 2b trials investigating petrelintide in subjects with overweight or obesity without T2D (ZUPREME-1) and with T2D (ZUPREME-2) are planned.

#### 3.4.3 Amycretin

Amycretin is a unimolecular dual agonist of the GLP1 and amylin receptors being developed as both a weekly subcutaneous injection and daily oral tablet. A 16-week phase 1 trial for the oral formulation reports a mean weight loss of 13% in the treatment group, compared to 1% for placebo (82). A phase 1b/2a trial of the weekly subcutaneous administration was conducted to assess safety, tolerability, pharmacokinetics and dose-related effects on body weight (83). Multiple dose titration arms were explored, ranging in duration from 20 to 36 weeks, with a mean weight loss of 24.3% at the highest dose of 60 mg at 36 weeks, compared to 1.1% in the placebo group.

#### 3.4.4 AZD6234

AZD6234 is a long-acting amylin analogue with superior affinity for amylin receptors over calcitonin receptors. A randomized, placebo-controlled SAD study in participants with a mean BMI 28.8 kg/m^2^ at doses of 0.3 mg, 0.9 mg, 1.5 mg, 2.7 mg, 3.0 mg, and 4.2 mg administered subcutaneously reports superior weight loss for all doses compared to placebo, although precise data have not yet been provided (84).

#### 3.4.5 GUBamy

GUBamy is a long-acting amylin RA administered weekly by subcutaneous administration. A 6-week phase 1 SAD study in lean and overweight participants shows a mean weight loss ranging 1.8% to 3.3% in the higher dose groups (3.5-6.0 mg), compared to weight gain of 1% for the placebo groups (85).

### 3.5 GLP-1/glucagon receptor agonists (oxyntomodulin analogues)

#### 3.5.1 Mazdutide

Oxyntomodulin is a proglucagon fragment that activates both GLP-1 and glucagon receptors. Mazdutide is an oxyntomodulin analogue administered subcutaneously once a week (86). A phase 1b clinical trial in Chinese individuals using a MAD study design shows a mean weight loss at 12 weeks of 11.7% at 9 mg, and 9.5% over 16 weeks at 10 mg, versus 1.8% and 3.3% in those who received placebo, respectively (86). Similarly, a 12-week phase 1b study in subjects with T2D demonstrates a mean weight loss of 5% and 5.4% at doses of 4.5 mg and 6.0 mg, respectively, compared to 0.9% with dulaglutide and 1.1% with placebo (87). However, this cohort included some individuals with normal weight.

In a 24-week phase 2 study investigating the effects of mazdutide on Chinese participants with overweight or obesity, there is a dose response effect, with a mean weight loss of 7.2%, 10.6%, and 11.6% at 3.0 mg, 4.5 mg, and 6.0 mg, respectively, compared to 1.0% with placebo (88). Reports of phase 2 data using 9 mg shows a placebo-adjusted mean weight loss of 15.4% after 24 weeks and 18.6% after 48 weeks (89). Another phase 2 study in subjects with poorly controlled T2D reveals mean HbA1c reduction of 1.4%, 1.7%, and 1.6% over 20 weeks at doses of 3.0 mg, 4.5 mg, and 6.0 mg, respectively, compared to a reduction of 1.4% with dulaglutide 1.5 mg and increase of 0.03% in the placebo group. Mean weight reductions corresponding to each group are 4.1%, 5.3%, 7.1%, 2.7%, and 1.4%, respectively (90). A 48-week phase 3 trial in Chinese subjects without T2D using mazdutide up to 6 mg reveals mean weight loss 12.6% compared to 0.5% weight gain for placebo (91).

#### 3.5.2 Pemvidutide

Pemvidutide is a dual GLP-1 and glucagon receptor co-agonist that is subcutaneously administered once a week. A small 12-week phase 1 trial in subjects with overweight or obesity, without T2D, using a MAD study design reveals a mean weight loss of 10.3% and 9.0% at the higher doses of 1.8 mg and 2.4 mg, respectively, compared to 1.6% in the placebo group (92). Data released from the MOMENTUM trial, a phase 2, 48-week study in subjects without T2D shows a mean weight loss of 10.3%, 11.2%, and 15.6% for the 1.2 mg, 1.8 mg, and 2.4 mg doses, respectively, compared to 2.2% for the placebo group (93). Another phase 1b trial evaluated adults with BMI ≥28 kg/m^2^ and T2D. Mean weight loss at 12 weeks is 4.4%, 6.1%, and 7.7% at 1.2 mg, 1.8 mg and 2.4 mg, respectively (93). A phase 1 pemvidutide fatty liver trial shows a significant mean reduction of liver fat at 12 weeks by 8.9%, 14.7%, and 11.3% corresponding to the 1.2 mg, 1.8 mg and 2.4 mg groups, compared to only 0.2% in the placebo group. Body weight reduction in this trial equates to 3.4%, 4.3%, and 3.7%, respectively, and 0.2% in the placebo group (94).

#### 3.5.3 Survodutide

Survodutide (formerly BI 456906) is another GLP-1 and glucagon receptor co-agonist administered via weekly subcutaneous injection. Two 16-week phase 1 trials, one in subjects with obesity and another with T2D, shows significant reductions in body weight and HbA1c, respectively (95). A mean weight loss of 13.7% is seen with a target dose of 2.4 mg biweekly and 9.0% with a target dose of 1.8 mg biweekly. A 46-week phase 2 study investigating the efficacy of survodutide in participants with BMI ≥27 kg/m^2^ without T2D demonstrates mean body weight losses of 6.8%, 13.6%, 16.7%, 18.7%, and 2.0% with 0.6 mg, 2.4 mg, 3.6 mg, 4.8 mg, and placebo, respectively (96). Another 16-week phase 2 trial in subjects with T2D and BMI 25–50 kg/m^2^ treated with survodutide 0.3, 0.9, 1.8, 2.7 mg weekly, 1.2 mg and 1.8 mg twice weekly, compared to semaglutide 1.0 mg weekly or placebo shows mean HbA1c percentage point reductions of 0.9, 1.5, 1.7, 1.6, 1.6, 1.7, 1.5, and 0.2, respectively, as well as mean body weight reductions significantly greater than placebo at all treatment doses, the highest being 8.7% for the 1.8 mg twice a week dose (97). Finally, a phase 2 study in subjects with metabolic dysfunction-associated steatohepatitis (MASH) and fibrosis at doses of 2.4 mg, 4.8 mg, and 6.0 mg, compared to placebo over 48 weeks, results in significant improvement of liver fat content and fibrosis, with mean weight reductions of 10%, 13%, 13%, and 0.7%, respectively (98). Approximately 40% of participants had T2D.

#### 3.5.4 Cotadutide

Cotadutide is a GLP-1 and glucagon receptor co-agonist administered subcutaneously once daily. In Asian participants with T2D over 10 weeks, a phase 1 trial investigating treatment with a target dose of 600 mcg, mean weight loss is 6.9% compared to 1.2% for placebo (99). A 41-day phase 2a study in participants with controlled T2D and BMI 27–40 kg/m^2^ shows a mean weight loss of 4.0% in those administered cotadutide with a target dose of 200 mcg daily, compared to 1.7% for the placebo group (100). In a phase 2b trial, subjects with BMI ≥25 kg/m^2^ and T2D were treated with cotadutide at doses of 100 mcg, 200 mcg and 300 mcg daily, compared to placebo and open-label liraglutide 1.8 mg. At 54 weeks, mean weight loss is 3.7%, 3.2%, and 5.0%, respectively, compared to 3.3% for the liraglutide group and 0.7% for placebo (101). Another 48-day phase 2a study in Japanese participants with uncontrolled T2D reveals mean weight loss of 2.1%, 3.3%, 3.3%, and 0.8% for the groups administered 100 mcg, 200 mcg, 300 mcg and placebo, respectively (102). Similarly, a 32-day phase 2a trial in subjects with BMI 25–45 kg/m^2^, T2D, and chronic kidney disease, at a target dose of 300 mcg daily, results in mean weight loss of 3.7% compared to 0.2% for placebo (103).

#### 3.5.5 Efinopegdutide

Efinopegdutide is a GLP-1 and glucagon receptor co-agonist administered subcutaneously once a week. A 24-week phase 2a study was conducted on participants with obesity and metabolic dysfunction-associated steatotic liver disease (MASLD) (104). About a third of them had T2D. The study design was a randomized open-label trial examining efinopegdutide 10 mg with an active comparator group administered semaglutide 1.0 mg. Mean weight loss is comparable, 8.5% and 7.1%, respectively. The mean reduction in liver fat content is 72.7% for the efinopegdutide group compared to 42.3% in the semaglutide group.

### 3.6 GLP-1/GIP/glucagon (triple G) receptor agonists

#### 3.6.1 Retatrutide

Retatrutide is a peptide triple agonist of GIP, GLP-1, and glucagon receptors administered subcutaneously once a week. A 48-week phase 2 study using a MAD strategy demonstrates a decrease in mean body weight of 8.7% with 1 mg, 17.1% with 4 mg, 22.8% with 8 mg, and 24.2% with 12 mg, compared to 2.1% with placebo (105). A second phase 2 study looking at the effects of retatrutide in patients with T2D and overweight or obesity finds that body weight decreases in a dose-dependent manner. At 36 weeks, the mean weight loss is 3.2% with 0.5 mg, 7.9% with 4 mg (initial dose 2 mg), 10.4% with 4 mg (no escalation), 16.8% with 8 mg (initial dose 2 mg), 16.3% with 8 mg (initial dose 4 mg), and 16.9% with 12 mg (initial dose 2 mg), versus 3.0% with placebo and 2.0% with 1.5 mg dulaglutide (106). Reductions in HbA1c at 36 weeks are significantly greater in all the retatrutide groups compared to placebo, except for the 0.5 mg group. HbA1c levels are also significantly lower in the 8 mg (initial dose 2 mg) and 12 mg groups compared to 1.5 mg dulaglutide. Finally, another 48-week phase 2 study in subjects with hepatic steatosis but not T2D results in a mean weight loss of 25.9% compared to 0.1% for placebo (107). Phase 3 trials for subjects with and without T2D are forthcoming (108, 109).

#### 3.6.2 UBT251

UBT251 is another GLP-1, GIP, and glucagon receptor triple co-agonist. A 12-week phase 1b trial using a MAD design in Chinese patients with overweight and obesity reveals a mean 15.1% weight loss at the highest target dose of 6 mg compared to a weight gain of 1.5% in the placebo group (110).

### 3.7 GLP-1/GLP-2 dual agonist

#### 3.7.1 Dapiglutide

Glucagon-like peptide 2 (GLP-2) is secreted from intestinal endocrine cells in response to nutrients. It improves intestinal barrier function (hence reducing systemic inflammation), delays gastric motility, and improves tolerance to GLP-1 agents (111). Dapiglutide, a co-agonist for GLP-1 and GLP-2 receptors, is being studied for anti-obesity therapy. A 4-week phase 1b MAD trial at weekly doses of 1.0 mg, 2.25 mg, 3.5 mg, and 6.0 mg, reports mean weight loss of 0.1%, 0.5%, 2.4%, and 4.5%, respectively (112). Placebo group weight loss is similar to the two lowest doses. In a 12-week phase 2a study, individuals with obesity were treated with weekly doses of 4 mg, 6 mg, or placebo. Mean weight loss is reported at 2.9%, 4.3%, and 2.2%, respectively (113).

### 3.8 Peptide YY

#### 3.8.1 CIN-110

CIN-110 is an analog of PYY, a gut hormone secreted in response to food intake, resulting in satiety and reduced energy consumption. A phase 1 SAD trial shows that after one dose of CIN-110, food consumption reduces by 28% and body weight by 1.8% at one week after administration (114).

## 4 Cytokines

### 4.1 CIN-109

**Figure 3:**
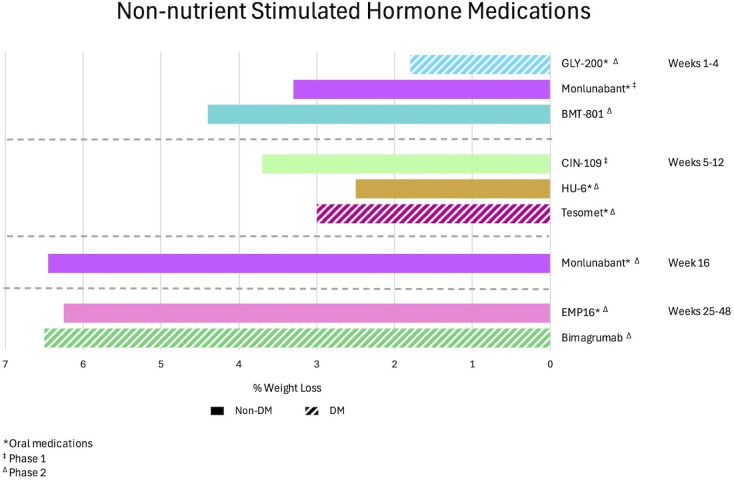
Observed weight loss of non-nutrient stimulated hormone anti-obesity medications in development organized by mean weight loss and duration of studies. DM, diabetes mellitus.

Growth differentiation factor 15 (GDF-15) is a member of the transforming growth factor beta (TGF-β) protein superfamily. There is evidence that it can promote satiety and reduce food intake via hindbrain pathways (115) and may also activate lipolysis (116). CIN-109, a GDF-15 analogue with prolonged elimination half-life administered subcutaneously, was examined in a phase 1 MAD, placebo-controlled study at weekly or biweekly dosing schedules. The weekly doses were administered at 5 mg, 10 mg, 15 mg, 20 mg, or 40 mg for 4–6 weeks, whereas the biweekly doses were given at 20 mg, 40 mg, and 60 mg for 8 weeks. The maximal weight loss is 3.7% for the 20 mg weekly dosing schedule (117). Notably, data presented for the 60 mg biweekly dosing demonstrates fat mass loss of >5% with loss of lean mass of <0.5%, indicating favorable body composition outcomes with treatment. **Figure 3** shows observed effectiveness for non-NuSH agents.

## 5 Endocannabinoid receptor antagonist

### 5.1 Monlunabant (INV-202)

Monlunabant is a peripherally acting cannabinoid receptor-1 (CB1) inverse agonist administered orally once a day. By selectively blocking CB1 receptors located outside the CNS, INV-202 aims to mitigate metabolic dysfunction without affecting the brain, thereby reducing potential neuropsychiatric side effects, which have plagued earlier generations of cannabinoid receptor blockers. A 28-day phase 1b trial results in mean weight loss of 3.3% at a dose of 25 mg, compared to a weight gain of 0.5% in the placebo group (118). A 16-week phase 2a trial is reported to result in mean weight loss of 6.4% in the 10 mg group compared to 0.6% in the placebo group. Limited additional weight loss is seen at higher doses of 20 mg and 50 mg (data not reported) (119).

## 6 Controlled metabolic accelerator

### 6.1 HU-6

Controlled metabolic accelerators (CMAs) are molecules that promote mitochondrial uncoupling at the level of the electron transport chain to increase energy expenditure and basal metabolic rate. HU-6 is a CMA, administered orally once a day, currently under development for the treatment of obesity and related complications, such as T2DM, MASH, severe hypertriglyceridemia, and heart failure with preserved ejection fraction (HFpEF) (120). An 8-week phase 2a trial studying liver fat and body weight reduction in patients with obesity and hepatic steatosis shows mean weight loss of 0.1%, 0.5%, 1.7%, and 2.5% for placebo, 150 mg, 300 mg, and 450 mg, respectively (121). Relative liver fat loss of approximately 27% to 36% is noted for the treatment groups, compared to a gain of 5% in the placebo group. Notably, the weight loss is predominantly fat, and minimal for skeletal muscle mass.

The HuMAIN study, a phase 2a trial examining HU-6 in subjects with HFpEF shows a mean weight loss of 3.1kg compared to 0.2 kg for the placebo group at three months (122). Other benefits include preferential loss of fat mass compared to lean mass, reductions in blood pressure, improvement in lipid parameters, and favorable changes in cardiac structure and function. The M-ACCEL trial is studying efficacy and safety in individuals with obesity and MASH (123).

## 7 Melanocortin 4 receptor agonists

### 7.1 Setmelanotide

Setmelanotide is a melanocortin-4 receptor (MC4R) agonist that is administered subcutaneously once a day (124). The melanocortin pathway in the hypothalamus is a primitive neuronal system that integrates peripheral signals into satiety, reduced energy consumption, and increased energy expenditure. Activation of pro-opiomelanocortin (POMC) neurons stimulate secretion of α-melanocyte stimulating hormone (α-MSH), the neurotransmitter that serves as a ligand for MC4R. MC4R-expressing neurons in hypothalamic nuclei then communicate with multiple brain systems to promote a negative energy balance. Many genes are involved directly and indirectly in the melanocortin system. Mutations in these genes are linked to monogenic obesity, characterized by early onset weight gain and hyperphagia. Pathogenic variants whose gene products function upstream of the MC4R could be bypassed with agonists for the receptor. In fact, setmelanotide is currently approved by the FDA to treat individuals with homozygous mutations in genes encoding for leptin receptor (LepR), POMC, and proprotein convertase subtilisin/kexin type 1 (PCSK1). It is also approved for patients with Bardet-Biedl Syndrome.

It is being investigated for treatment of various heterozygous mutations in LepR, POMC, or PCSK1 genes, and homozygous or heterozygous mutations in the steroid receptor coactivator-1 (SRC1) or SH2B adapter protein 1 (SH2B1) genes (125). Another study is examining the safety and efficacy of setmelanotide in individuals with other variants not previously examined (126). Preliminary data from the DAYBREAK Trial reveals a mean weight loss of 12.4% in subjects treated continuously for 40 weeks, comprising a group of composite variants, including pleckstrin homology domain-interacting protein (PHIP), Plexin-A (PLXNA)1-4, semaphorin 3 (SEMA3[A-D, F, G]), single-minded homolog 1 (SIM1), MAGE family member L2 (MAGEL2), and retinosa pigmentosa GTPase regulator interacting protein 1 like (RPGRIP1L) genes. Given the rarity of these mutations, sample sizes of individual variants are too small to make firm conclusions.

**Figure 4:**
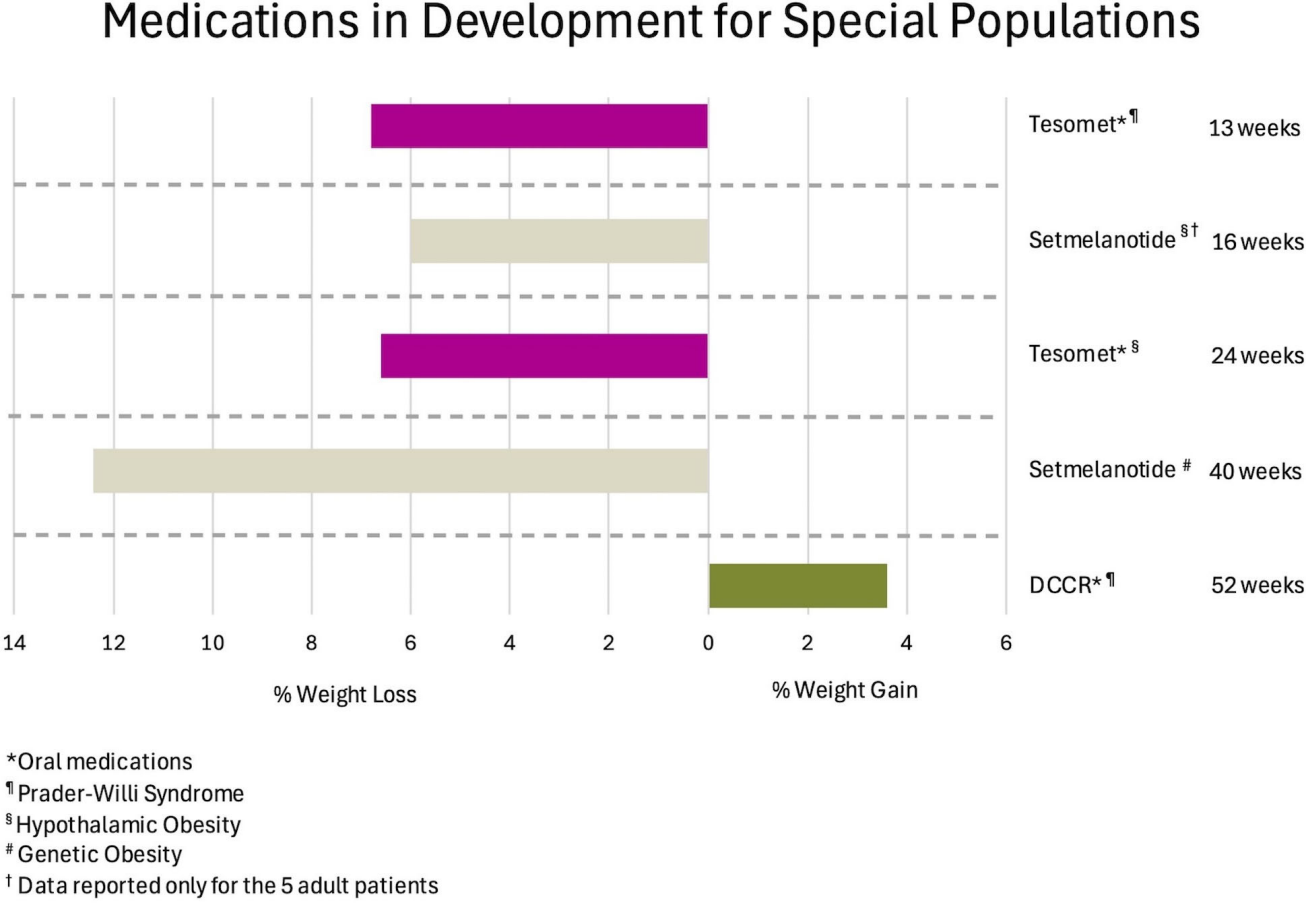
Observed weight loss of anti-obesity medications in development for special populations, organized by mean weight change and duration of studies. DCCR, Diazoxide Choline Extended Release.

Setmelanotide is currently being studied for treatment of acquired hypothalamic obesity (HO), a condition occurring after injury to the relevant satiety centers, also characterized by hyperphagia and rapid weight gain. A phase 2, 16-week, open-label study in 18 subjects ranging from 6 to 40 years of age results in a mean BMI reduction of 18% in those younger than 18 years, and 6% for those 18 years or older, accompanied by significant reduction of hunger scores (127). **Figure 4** depicts outcomes for therapies in special populations, such as those with genetic obesity, Prader-Willi Syndrome (PWS), and HO.

### 7.2 Bremelanotide

Bremelanotide is a selective MC4R agonist administered subcutaneously once a day. BMT-801 is a combination of bremelanotide and tirzepatide, a unimolecular peptide agonist of GLP-1 and GIP receptors. A phase 2 study randomized participants to bremelanotide 1.25 mg daily and tirzepatide 2.5 mg weekly, bremelanotide alone, tirzepatide alone, and placebo, all for 4 weeks, after a 4-week run in period with tirzepatide 2.5 mg weekly (128). At the end of the trial, mean weight loss of 4.4% is reported in the combination group, compared to 1.6% for placebo. Interestingly, the group that transitioned from tirzepatide to bremelanotide shows maintenance of lost weight.

## 8 Myostatin and activin receptor antagonists

### 8.1 Garetosmab and trevogrumab

Stimulation of receptors for myostatin and activin inhibit skeletal muscle growth (129–131). Garetosmab and trevogrumab are monoclonal blocking antibodies that bind to activin A and myostatin receptors, respectively, to reduce muscle catabolism (132, 133). Myostatin and activin receptor blockade is being developed as a strategy to mitigate the loss of skeletal mass that accompanies weight loss through lifestyle, pharmacologic and surgical therapies. A phase 1 trial in healthy male and postmenopausal female volunteers was conducted using single dose and multiple dose groups (134). After a single combined intravenous (IV) dose of trevogrumab 6 mg/kg and garetosmab 10 mg/kg, an increase in thigh muscle volume (TMV) of 7.7% is seen at 8 weeks, and 4.6% increase with trevogrumab monotherapy, both statistically significant compared to placebo. The combination group shows a statistically significant decrease in total fat mass and android fat mass (AFM) of 4.6% and 6.7%, respectively.

In the multiple dose groups, females and males received garetosmab every 4 weeks for 4 doses, and a group of females were treated with combination therapy every 2 weeks, all with 3 doses. In the combination group, TMV increases after 3 doses, but returns to similar levels as placebo after 28 weeks. A pooled analysis of all groups shows reductions of 14.3% and 20.1% in AFM and visceral fat, respectively.

### 8.2 Bimagrumab

Bimagrumab is a human monoclonal antibody that inhibits activin type II receptors (ActRII), stimulating skeletal muscle growth (135). In a small study of subjects who were overweight and insulin resistant, a single IV infusion causes loss of fat mass, increased lean mass, and improved insulin sensitivity (136). A 48-week phase 2 trial studying the effects of IV bimagrumab in individuals with T2D and BMI 28–40 kg/m^2^ finds that total body fat mass decreases 20.5% (*vs* 0.5% for placebo), lean mass increases 3.6% (*vs* a decrease of 0.8% for placebo), waist circumference decreases 9 cm (*vs* 0.5 cm for placebo), HbA1c level decreases 0.8% (*vs* 0.04% increase for placebo), and body weight decreases 6.5% (*vs* 0.8% placebo) (135). The overall weight loss is modest, but preclinical studies show a possible synergistic effect when combined with semaglutide or tirzepatide (137). Human studies combining bimagrumab with tirzepatide (138) or semaglutide (139) are in progress.

## 9 Centrally acting psychoactive agents

### 9.1 Tesomet (tesofensine and metoprolol)

Tesofensine is a daily oral centrally acting reuptake inhibitor of norepinephrine, dopamine, and serotonin, resulting in weight reduction (140). A 24-week phase 2 trial of tesofensine results in a mean weight loss of 4.5%, 9.2% and 10.6% at doses of 0.25 mg, 0.5 mg, and 1.0 mg, respectively, all compared to 2.0% for the placebo group (140). Because of observed effects on heart rate and blood pressure, metoprolol is added to increase its cardiac safety profile (141, 142). A 90-day randomized placebo-controlled trial in participants with obesity and T2D investigating tesofensine 0.5 mg with metoprolol 100 mg daily shows a mean weight loss of 3% compared to 1% for placebo, along with a significant reduction in resting heart rate compared to the placebo group, and a trend toward reduction in systolic and diastolic blood pressure (143).

PWS is a rare condition characterized by severe early onset obesity, hyperphagia, and developmental delay. There are no highly effective treatments for the hyperphagia and obesity. A small study examining the effects of tesomet in adults with PWS was initiated with six subjects in the treatment arm and three in the placebo arm. There was a high rate of discontinuation due to adverse effects. At 13 weeks, only two participants remained in each arm, with observed mean weight loss of 6.8% for those receiving tesomet, compared to 0.8% for those on placebo (144). It was unexpectedly observed that plasma levels for the drug were significantly elevated compared to individuals without PWS, potentially explaining the high number of dropouts. Notably, using a validated hyperphagia questionnaire, scores decrease from 10.0 to 0, and 11.7 to 9.5, respectively. There are no significant differences in blood pressure or heart rate between those receiving tesomet or placebo.

A phase 2 randomized placebo-controlled trial examined tesomet in adults with HO. After 24 weeks, a mean weight loss of 6.6% in the treatment group and 0.3% in the placebo group are observed. Satiety scores are higher for the tesomet group at week 16 but return to levels equal to the placebo group by the end of the study (145).

### 9.2 Diazoxide choline extended-release (DCCR)

DCCR is an activator of potassium channels in Neuropeptide Y/Agouti related-protein (NPY/AgRP) neurons, which are responsible for appetite stimulation. Activation of membrane channels leads to reduction of peptide secretion in these cells (146). A phase 3 study in subjects ≥4 years of age and PWS do not show significant changes in body weight at 13 weeks, although lean body mass/fat mass ratio increase significantly in the treatment group (147). An open-label extension study 52 weeks from completion of the phase 3 trial shows 3.6% mean weight gain, but it was almost all attributable to an increase in lean mass (147). Hyperphagia scores decrease significantly in the extension phase. Of note, between the time of initial submission of the present manuscript and publication, DCCR was approved by the FDA for the treatment of hyperphagia in patients with PWS (148).

## 10 Non-absorbed gastrointestinal agents

### 10.1 GLY-200

GLY-200 is a non-absorbed, orally administered mucin-complexing polymer that increases the mucus barrier in the duodenum (149). Through mucosal exclusion of the foregut, GLY 200 works by reproducing and mimicking the metabolic effects of bariatric surgery. In a 5-day phase 1 MAD trial where four groups of healthy volunteers received twice-daily or thrice-daily doses of GLY-200, decreases in glucose and insulin, along with increases in bile acids, GLP-1, peptide YY, and glicentin are noted in the cohorts with twice-daily dosing versus those receiving placebo (150). A phase 2 trial in subjects with T2D investigating 2 g twice daily over 14 days reveals statistically significant improved glycemic control compared to placebo, which correlates with a mean weight loss of -1.8% (150–152).

### 10.2 EMP16 (orlistat-acarbose)

EMP16 is an oral medication that combines acarbose and orlistat, administered orally three times a day with meals. Acarbose is a glucosidase and amylase inhibitor, which delays carbohydrate digestion in the GI tract. Orlistat is a lipase inhibitor that blocks dietary fat absorption (153). A 26-week randomized controlled trial shows mean weight loss of 6.3%, 5.5%, and 0.8% at target doses of 150 mg/50 mg, 120 mg/40 mg, and placebo, respectively (154).

## 11 Discussion

Obesity and related complications are stubborn conditions that defy long-term improvement with lifestyle interventions alone (7). Until recently, bariatric surgery was the only therapy to produce significant durable weight loss and amelioration of metabolic comorbidities. The recent appearance of highly effective AOMs, semaglutide and tirzepatide, heralds a new era in the treatment of obesity. Beyond weight loss, data are emerging for the undeniable benefits on a wide variety of maladies, including cardiovascular disease (155–157), obstructive sleep apnea (158), MASLD (159), addiction disorders (160, 161), and dementia (162). In people with prediabetes, both medications have shown remarkable effects on glycemic control (163, 164). There is also observational evidence that GLP-1 RAs may protect against obesity-associated cancers (165).

Despite these promising benefits, unmet needs remain. Not all patients can tolerate current therapies, mostly due to adverse GI symptoms. Some individuals have contraindications to incretin-based medications, such as clinically significant gastroparesis or history of pancreatitis. Because of heterogeneity of response, some patients may exhibit suboptimal weight loss. In fact, at one year after initiation, only 20-50% of patients may remain on therapy (166, 167). Aside from adverse effects, other reasons that have been implicated for real-world discontinuation include perceived or realized suboptimal effectiveness, cost and access challenges, and socioeconomic inequalities (167). Moreover, the expected weight reduction in those with extreme obesity may be insufficient to meet all desired health outcomes and goals. HCPs and patients should not lose sight that AOMs are adjuncts to optimal lifestyle behaviors. In modern high volume, fast paced clinic settings it is tempting to rely on pharmacotherapy as the sole intervention. Some patients may need extra support for the continuance of healthy lifestyle behaviors, even with long-term use of pharmacologic agents. Finally, the high cost and lack of reimbursement for many patients limits access to these therapies for considerable numbers that could benefit. As many of these nascent molecules reach the market, the hope is that economic forces will drive down prices.

Even if these challenges are addressed, questions remain. For example, as multiple agents with similar MOA, efficacy, and safety enter an increasingly crowded market, how will each of them be positioned to address specific patient phenotypes? For patients with T2D, perhaps duration of their disease and degree of glycemic control will dictate choice of therapy based on effectiveness for HbA1c reduction. Should patients with cardiovascular disease be treated with drugs that have proven morbidity and mortality benefit, or are these outcomes a class effect? Will AOMs that target glucagon receptors be superior for reversing liver fibrosis? For which patients would an amylin receptor agonist be the first choice? Are there clinical outcomes for which the use of muscle preserving therapies are desired? And if so, which patients would benefit the most? Much research will be needed to answer these questions, both from prospective randomized controlled trials and real-world observational studies.

Similarly, beyond targeting multiple receptors with one molecule, more work is needed to assess enhanced efficacy and safety of combining drugs with divergent MOA. As previously noted, the pharmaceutical industry has already begun to examine possible synergistic strategies, such as targeting receptors for NuSH’s and the melanocortin system, or incretin therapies with myostatin and activin receptor antagonists. Likewise, would CMAs safely push weight loss and metabolic benefits beyond the plateau commonly experienced with existing AOMs? Since effectiveness of cannabinoid receptor antagonists is limited by brain penetration and neuropsychiatric side effects, perhaps using lower doses or agents with less access to the CNS in combination with NuSH mimetics would result in superior outcomes. Finally, there is much to learn for how best to combine emerging therapies in bariatric surgery patients with insufficient weight loss or recurrent weight gain. Many unanswered issues remain, including choosing the best agent, timing of initiation after a procedure, appropriate monitoring for vitamins and micronutrients, and knowing when weight loss may be excessive. Just a few years ago, these questions were not being asked because our pharmacologic therapies were very limited.

Despite the pleiotropic benefits increasingly observed for existing novel AOMs, HCPs and patients should temper enthusiasm with potential long-term adverse effects, and costs to society and healthcare systems. This is especially true if overweight and obesity are excessively medicalized, leading to overutilization of expensive treatments, including current and emerging pharmacotherapies (168). The *Lancet Diabetes & Endocrinology* Commission on the Definition and Diagnosis of Clinical Obesity aims to refine definitions of excess adiposity to help prioritize therapeutic interventions and public health policies (169).These efforts are quite relevant as economic analyses have challenged the cost effectiveness of novel agents despite their efficacy and benefits (170). In the United Kingdom, the National Institute for Health and Care Excellence (NICE) has provided guidance to the National Health Service (NHS) regarding cost effective use of novel AOMs, prioritizing patients with higher BMI and related complications, thereby limiting access for those with less complicated obesity (171). Additionally, given the tremendous increase in utilization of incretin hormone medications, unforeseen adverse events may emerge that were not identified in the pivotal clinical trials, such as psychiatric events, hair loss, and ocular complications (172). Because they cause C-cell hyperplasia in rodent models, GLP-1 based drugs are contraindicated in patients with personal or family histories of medullary thyroid cancer or multiple endocrine neoplasia type 2. Large observational studies show mixed results with respect to associations between use of GLP-1 medications and incidence of thyroid cancer (173, 174), without proven causality in humans. Preclinical studies have indicated that chronic activation of GLP-1 receptors may induce dysplastic pancreatic lesions (175), but large human datasets have not shown a causal relationship between GLP-1 RAs and pancreatic cancer (173, 176).

This report has significant limitations that should be considered. Given the aim of introducing the reader to novel AOMs in the pipeline, much of the data are limited by small sample sizes and early stages of development. As such, due to the narrative nature of the present review, it lacks systematic control for multiple biases and the power of a meta-analytic approach. Because of incongruent phases of development and heterogeneous study designs, it is impossible to compare efficacy, safety, and tolerability between the reported agents. It is also possible other nascent molecules may have been overlooked if they were not identified by our search strategy.

Nevertheless, the medical profession should carefully embrace the new era of treating excess adiposity and related complications. As our therapeutic toolbox grows, the horizon appears bright for people living with obesity and metabolic diseases.

## 12 Conclusion

Safe and effective treatments for obesity have lagged behind the relentless epidemic of excess adiposity. In the last few years, highly effective and safe AOMs have appeared for clinical use, a culmination of decades of work by many to better understand the complexity of human weight regulation. We are starting to witness the fruits of that work with dozens of agents in development that will be more efficacious for obesity and its related complications. As they emerge, research is needed to learn how best to use them in combination with each other and with other modalities. The field is primed for optimizing precision approaches for the benefit of patients suffering with chronic metabolic disease.
